# Editorial: XVII SOLANACEAE2022 meets the 2020 decade challenges

**DOI:** 10.3389/fpls.2025.1570346

**Published:** 2025-03-10

**Authors:** Ifigeneia Mellidou, Antonio Granell, Hiroshi Ezura, Panagiotis Kalaitzis, Nathalie Gonzalez, Alain Goossens, Mondher Bouzayen, Angelos K. Kanellis

**Affiliations:** ^1^ Institute of Plant Breeding and Genetic Resources, Hellenic Agricultural Organization – DEMETER, Thessaloniki, Greece; ^2^ Instituto de Biología Molecular y Celular de Plantas (IBMCP), Consejo Superior de Investigaciones Científicas (CSIC), Universitat Politècnica de València, València, Spain; ^3^ Institute of Life and Environmental Sciences, University of Tsukuba, Tsukuba, Japan; ^4^ Department of Horticultural Genetics and Biotechnology, Mediterranean Agronomic Institute of Chania, Chania, Greece; ^5^ National Research Institute for Agriculture, Food and Environment (INRAE), UMR1332 Biologie du Fruit et Pathologie, Université Bordeaux, Villenave d’Ornon, France; ^6^ Department of Plant Biotechnology and Bioinformatics, Ghent University, Ghent, Belgium; ^7^ VIB Centre for Plant Systems Biology, Ghent, Belgium; ^8^ Laboratoire de Recherche en Sciences Végétales, Equipe Génomique et Biotechnologie des Fruits, UMR 5546, Centre national de la recherche scientifique (CNRS), Paul Sabatier (UPS), Toulouse Institut National Polytechnique (INP), Université de Toulouse, Toulouse, France; ^9^ Group of Biotechnology of Pharmaceutical Plants, Laboratory of Pharmacognosy, Department of Pharmaceutical Sciences, Aristotle University of Thessaloniki, Thessaloniki, Greece

**Keywords:** *Solanaceae*, cultivation, postharvest physiology, fruit quality, fruit ripening

Given the ever-increasing world population and the striking losses of plant productivity due to global warming, new breeding strategies are necessary to fortify stress resilience of crops (He et al., 2018). The International Conference XVII SOLANACEAE2022 on the Plant Family of *Solanaceae* took place in Thessaloniki, GREECE, on November 1-5, 2022, and attracted more than 200 distinguished researchers and academics from more than 20 countries. The present Research Topic hosts a series of current research studies that are relevant for *Solanaceae* species, on the mechanisms underlying different phases of fruit development, ripening, postharvest potential, and stress-responses.

Fruit development is a critical process for plants that usually starts with fertilization (Quinet et al., 2019). Parthenocarpic fruits, however, can be produced independently of fertilization, and parthenocarpy is considered a highly desirable agronomic trait. The study of Tran et al. on pollination, pollen tube growth and fertilization highlighted the intricate signaling pathways and cellular mechanisms that regulate tomato fruit set and development. By using different types of pollen, the authors reveal that full penetration of pollen tubes can trigger fruit set and early development without need for fertilization, as evidenced by parthenocarpic fruit formation, most likely by activating the transcriptomic reprogramming that leads to initiation and progress of fruit development.

Fruit set is an essential transition process from opening flower to developing fruit, triggered by sophisticated interactions between multiple hormonal signals and diverse transcriptional alterations (Ezura et al., 2023). By combining genome-wide transcriptomic profiling and ChIP-seq analysis, Li et al. (2025) demonstrated that both pollination-dependent and -independent (induced by auxin treatment) fruit set triggered the expression of a large set of genes, that are mainly expressed in maternal tissues and related to auxin, gibberellin, brassinosteroid and ethylene signaling. This transcriptional reprogramming correlates with the dynamic changes in H3K9ac and H3K4me3 histone marks regardless of the type of fruit set.

Fruit ripening is a complex process inducing alterations in fruit texture and color involving cell-wall metabolic processes. During ripening, cell wall assembly can be modified by changes in the structure and distribution of arabinogalactan proteins (AGPs), that are present in the extracellular matrix (Kutyrieva-Nowak et al., 2023). Using transgenic tomato lines with modified prolyl-4-hydroxylase (*P4H3*) gene expression, Kutyrieva-Nowak et al. demonstrated that changes in P4H3 activity significantly affected AGP content and structure during fruit ripening, resulting in altered cell-wall composition and stability. Silencing *P4H3* reduced AGP content and delayed fruit ripening, while overexpression enhanced AGP levels and accelerated ripening, highlighting the critical role of AGPs and *P4H3* in regulating cell-wall dynamics and fruit softening.

Cold storage is a common method employed to prolong the postharvest life of tomatoes, but this practice can also trigger undesirable chilling injury symptoms, which contribute to extensive fruit losses (Albornoz et al., 2022). Mitalo et al. conducted a comprehensive transcriptome analysis of the effect of an inhibitor of ethylene perception 1-methylcyclopropene (1-MCP), on fruit ripening and chilling injury during tomato storage. Their study revealed that cold stress can trigger a large-scale and unique transcriptome adjustment, associated with active ethylene biosynthesis and signaling, ribosome biogenesis, DNA methylation, chromatin remodeling, and alternative splicing events.

Genetic factors have a major impact on tomato postharvest potential. Notwithstanding the large genetic erosion in the gene pool among modern cultivars, some landraces exhibiting excellent nutritional and organoleptic properties and post-harvest behavior can be still found in local markets (Blanca et al., 2022; Pons et al., 2022). Mellidou et al. investigated the postharvest life and nutritional quality of 130 Greek tomato accessions, using metabolomics and transcriptomics. Distinct differences in flavor and nutritional compounds between short shelf-life (SSL) and long shelf-life (LSL) cultivars were emphasized and correlated with the up-regulation of key genes involved in cell wall synthesis, polyamine synthesis, and ABA catabolism in LSL cultivars, while those related to ethylene biosynthesis and cell-wall degradation were stimulated in SSL cultivars.

Apart from traditional varieties, unravelling the reproductive bottlenecks present in the *Lycopersicon* clade caused by tomato domestication would facilitate the use of wild relative germplasm in plant breeding (Muñoz-Sanz et al., 2020). The reproductive barriers within the tomato clade were examined by Moreels et al., focusing on self-incompatibility (SI) and unilateral incompatibility (UI). The review described the *modus operandi* of gametophytic and sporophytic SI and explored the genetic regulation involving S-RNases and F-box proteins. The paper also discussed the SI-to-self-compatible transition in different tomato species and the role of UI in preventing interspecific crosses.

In the context of global warming, breeding for heat stress tolerance is becoming of utmost importance (Han et al., 2021). The utilization of wild crops may provide the means to introduce heat tolerance traits into modern varieties, as highlighted in Bollier et al. Transcriptome profiling of floral buds in a large set of tomato cultivars identified specific genes that contribute to heat tolerance, such as those encoding heat shock proteins, reactive oxygen species scavengers and transporters that maintain cellular integrity under stress.

Among tomato wild species, *S. pimpinellifolium* is considered as an attractive source for breeding, with several accessions being characterized as salt-tolerant (Rao et al., 2013). Bashary et al. explored the development of heat stress-tolerant tomato cultivars obtained by backcrossing inbred lines derived from a cross between *S. pimpinellifolium* and *S. lycopersicum.* The study identified a 0.8 Mb region on chromosome 9 common to the thermotolerant lines, containing several candidate genes linked to heat stress response, including genes related to chaperonin 60 and ABC transporters, providing a valuable genetic reservoir for the breeding of more heat-resilient tomato varieties.

In nature, plants typically encounter a combination of stresses such as concomitant heat and salinity (Nadeem et al., 2022). David-Rogeat et al. investigated the effect of these combined stresses on African eggplant (*Solanum aethiopicum* L.) cultivars. Combination of stresses can have synergistic, antagonistic, or additive effects, making it complicated to establish an adequate plant response. High temperatures reduced leaf biomass while cell membrane stability was reduced by salinity. Although heat stress alone affected pollen viability and fruit set, the combination with salinity exacerbated these effects, leading to a greater drop in yield. Understanding these interactions is crucial for breeders to determine if a tolerant crop can withstand a combination of stresses.

Modern genomic tools have revolutionized plant stress research, providing detailed insights into the genetic and molecular basis of stress responses. Zagorščak et al. developed a unified potato genome model (Hoopes et al., 2022; Bozan et al., 2023), UniTato, by combining annotations from all the existing gene models, offering a useful platform, accessible via an Apollo web interface, to explore genetic variations and identify candidate genes associated with stress tolerance. Such resources are invaluable for breeding programs, enabling the selection of desirable traits with greater precision and efficiency.
